# Theory and practice gap in teaching nursing students and associated factors at teaching institutions: Mixed study

**DOI:** 10.1016/j.jtumed.2026.07.014

**Published:** 2026-08-13

**Authors:** Samuel Abate Walda, Abebe Dechasa, Meseret Robi Tura, Keneni Dina

**Affiliations:** Department of Nursing, College of Health Sciences and Referral Hospital, Ambo University, Ambo, Ethiopia

**Keywords:** التطبيق، النظرية, الفجوة بين النظرية والتطبيق، تعليم التمريض, التدريب السريري, Practice, Theory, Theory–practice gap

## Abstract

**Background:**

Nursing education involves various teaching and learning techniques, as well as sharing information, wisdom, and sound judgment, aiming to close the knowledge–practice gap and reaching the ultimate goal of improving quality of care and benefiting the population served.

**Objectives:**

This study aimed to assess the theory–practice gap in teaching nursing students and associated factors at teaching institutions in west and southwest Shewa zones, Oromia, Ethiopia during 2024.

**Methods:**

An institutional-based explanatory sequential cross-sectional study was conducted using both quantitative and qualitative approaches from June to August 2024. In total, 422 undergraduate nursing students participated in the quantitative component using a structured self-administered questionnaire, and 16 nursing instructors were purposively selected for in-depth interviews. Quantitative data were analyzed using descriptive statistics and binary logistic regression, whereas qualitative data were analyzed by thematic analysis, with triangulation applied to integrate the findings.

**Results:**

Almost all participants (99.1%) reported the presence of a theory–practice gap, with an overall mean score of 3.6. Students who practiced in non-teaching hospitals were significantly more likely to experience the theory–practice gap than those in teaching hospitals (adjusted odds ratio = 2.83; 95% confidence interval: 1.43–5.60). The qualitative findings identified several contributing factors, including excessive emphasis on theoretical teaching, limited clinical integration, inadequate communication between theoretical and clinical instructors, insufficient learning resources, and weak institutional collaboration between universities and healthcare facilities.

## الملخص

**أهداف البحث:**

يُعد التكامل بين التعليم النظري والتدريب السريري من الركائز الأساسية في تعليم التمريض، إذ يسهم في تمكين الطلبة من تطبيق المعارف المكتسبة في المواقف السريرية وتحسين جودة الرعاية وسلامة المرضى. هدفت هذه الدراسة إلى تقييم حجم الفجوة بين النظرية والتطبيق في تعليم طلبة التمريض، وتحديد العوامل المرتبطة بها، واستكشاف تجارب الطلبة والمدرسين في مؤسسات التعليم العالي الواقعة في منطقتي غرب وجنوب غرب شوا بإقليم أوروميا في إثيوبيا.

**طرق البحث:**

أُجريت دراسة مقطعية مؤسسية باستخدام تصميم مختلط تفسيري متتابع خلال الفترة من يونيو إلى أغسطس 2024. شمل الجزء الكمي 422 طالباً وطالبة من طلبة التمريض في مرحلة البكالوريوس، جرى اختيارهم باستخدام التوزيع النسبي والعينة العشوائية البسيطة، وجُمعت البيانات بواسطة استبانة ذاتية منظمة. كما شمل الجزء النوعي 16 مدرساً للتمريض تم اختيارهم قصديّاً لإجراء مقابلات معمقة. حُللت البيانات الكمية باستخدام الإحصاءات الوصفية والانحدار اللوجستي الثنائي، في حين حُللت البيانات النوعية باستخدام التحليل الموضوعي، ثم دُمجت النتائج عن طريق التثليث.

**النتائج:**

أفاد معظم المشاركين، بنسبة 99.1%، بوجود فجوة بين النظرية والتطبيق، وبلغ المتوسط العام لدرجة الفجوة 3.6. وكان الطلبة الذين تلقوا تدريبهم في مستشفيات غير تعليمية أكثر عرضة بدرجة معنوية لتجربة هذه الفجوة مقارنة بالطلبة المتدربين في المستشفيات التعليمية، بنسبة أرجحية معدلة بلغت 2.83 وفاصل ثقة 95% من 1.43 إلى 5.60. كما كشفت النتائج النوعية عن عدد من العوامل المساهمة، منها الإفراط في التركيز على التدريس النظري، وضعف دمج التدريب السريري بالمحتوى الأكاديمي، وقصور التواصل بين المدرسين النظريين والمشرفين السريريين، ونقص مصادر التعلم والتجهيزات، وضعف التعاون المؤسسي بين الجامعات والمرافق الصحية. كذلك أشار المشاركون إلى تفاوت الخبرات السريرية، ومحدودية مشاركة أعضاء هيئة التدريس في بيئات التدريب، وضعف الإعداد السريري والثقة لدى الطلبة.

**الاستنتاج:**

تكشف هذه الدراسة عن وجود فجوة كبيرة بين التعليم النظري والممارسة السريرية في برامج التمريض بالمؤسسات المشمولة. ويتطلب تقليص هذه الفجوة تعزيز الشراكة بين الجامعات والمستشفيات، وزيادة أعداد المدرسين السريريين المؤهلين، وتحسين التواصل والتنسيق بين التدريس النظري والسريري، وتوفير مصادر تعلم وتجهيزات مناسبة، إلى جانب تطوير المناهج لتشمل التعلم القائم على الكفايات والمحاكاة والحالات السريرية والتعرض المبكر والمنظم للبيئة الإكلينيكية. وقد تسهم هذه الإجراءات في تحسين كفاءة الخريجين وجودة تعليم التمريض وسلامة رعاية المرضى.

## Introduction

The theory–practice gap is defined as the difference between hypothetical practice and common practice, and the difference between taught general principles and the difficulty in interpreting them for application to a specific situation. In particular, it is the gap between taught abstract nursing theory and its use in practice in nursing education.^1^

According to the Oxford English Dictionary, a theory is a series of statements or principles developed to explain a collection of facts or occurrences, particularly one that has been put to the test time and time again, is generally accepted, and can be used to forecast natural phenomena. Practice refers to an action or a process that is used to perform an action. These terms have opposing definitions but when considered in the context of a professional setting, they must facilitate putting theory into practice.^2^

Nursing education has a theoretical component and clinical component. Theoretical elements support the knowledge and insights provided in the classroom^3^ as a framework to allow nursing students to comprehend the profession's history, the human body, diseases, and nursing techniques. The aims of the clinical learning component are to enhance clinical nursing abilities and characteristics.^4^

The clinical learning component is essential for assessing a nursing student's performance in the clinical setting and giving students the opportunity to hone their abilities, forge their professional identities, learn more, and apply their theoretical and practical knowledge in the clinical setting.^5^

The three pillars of nursing are practice, research, and theory. The interactions between these three pillars are reciprocal and cyclical. Clinical practice generates knowledge to support theory as well as research topics. Our practice is influenced by research, and growth of theory furthers our understanding. Research and practice are improved by theory. Applying knowledge, skills, compassion, and artistic ability to provide patients with effective, efficient, and thoughtful care is a necessary component of excellent nursing practice. Research findings have contributed significantly to knowledge that is utilized to guide clinical nursing decisions. All decisions regarding patient treatment should ideally be supported by research findings. A protocol is created using research findings, and then applied to everyday nursing practice.^6^

The theory–practice gap is highly relevant because it endangers patient safety, and high adherence rates to guidelines are difficult to achieve if there is a gap between theory and practice. Nursing implementation programs should be well planned and consider the features of the target group, and the social and organizational context. Nursing theory is of crucial importance for helping healthcare workers to understand the rationale that underlies guidelines.^7^

The theory–practice gap is the difference between what nurses learn about theoretical nursing concepts in the classroom and what they actually encounter on practical rotations^8^ in clinical placement (i.e., nursing practice).^9^

In nursing education, the theoretical component has begun to gain ground, where new programs and subspecialists are being created, and the emphasis on clinical education has reduced as a consequence. In addition, nursing educators have started to place more emphasis on theory and research than practical competencies. Due to placing clinical education second to prioritizing services, the existing curriculum content and clinical experiences fail to meet the needs of learners in terms of clinical settings.^5^

The best care plans can be written by nurses who have strong understanding of theory, but they may find it difficult to put their knowledge of pathophysiology and treatment strategies into practice^10^ Conversely, nurses with strong clinical practice abilities frequently find it challenging to justify care in terms of theory.^11^

The theory–practice gap also affects the motivation, confidence, and professional identity of nurses. Studies have shown that inconsistencies between classroom teaching and clinical experience can lead to confusion, stress, and reduced preparedness for clinical practice,^10^ where the contributing factors include educational content, clinical exposure, institutional support, and organizational challenges.^11^

Therefore, understanding the magnitude of the theory–practice gap and any contributing factors is essential for improving nursing education and strengthening clinical practice systems.

## Rationale for the study

Despite continuous improvements in nursing curricula and educational strategies, a persistent gap between theoretical instruction and clinical practice remains a major challenge in nursing education. This theory–practice gap limits the ability of students to apply classroom knowledge in real clinical settings, weakens clinical judgment, reduces professional confidence, and compromises the quality and safety of patient care. The gap is further widened by inadequate clinical exposure, the shortage of skilled clinical instructors, weak coordination between academic institutions and healthcare facilities, and limited learning resources.

Several international studies have documented the existence of the theory–practice gap, but limited empirical evidence is available for Ethiopia, particularly in the west and southwest Shewa Zones. The lack of localized data makes it difficult for educators, policymakers, and health institutions to design effective strategies tailored to the nursing education context in Ethiopia. Therefore, generating context-specific evidence is essential to guide curriculum reform, strengthen clinical teaching, improve institutional integration, and enhance the overall quality of nursing education and patient care.

## Objectives of the study

The objectives of this study were to assess the extent of the theory–practice gap in nursing education, identify factors associated with the gap, and explore how the gap is experienced by students and instructors in selected higher education institutions in the west and southwest Shewa Zones, Oromia, Ethiopia.

## Materials and Methods

### Study design and setting

This institutional-based explanatory sequential cross-sectional study was designed using quantitative and qualitative approaches, and conducted from June 1 to August 30, 2024, in selected public and private higher education institutions in the west and southwest Shewa Zones, Oromia, Ethiopia. The study followed an explanatory sequential mixed-method design, where quantitative data were collected and analyzed first, before conducting qualitative data collection to explain and elaborate on the quantitative findings.

### Study population

The quantitative population consisted of all undergraduate nursing interns enrolled in the selected institutions during the study period. The qualitative population included nursing instructors actively engaged in theoretical and/or clinical nursing teaching.

### Sample size and sampling procedure

The quantitative sample size was 422 students, which was determined using a single population proportion formula with an assumption of 50%, 95% confidence level, and 10% non-response rate. Participants were selected using proportional allocation across institutions, followed by simple random sampling.

For the qualitative component, 16 nursing instructors were selected using purposive sampling based on their teaching experience and involvement in both theoretical and clinical instruction. Data collection was continued until saturation, which was defined as the point at which no new themes emerged.

### Data collection tools and procedures

Quantitative data were collected using a structured, self-administered questionnaire adapted from a previously validated instrument used in a study conducted at the University of Lahore, Lahore, Pakistan.^12^ The tool was contextualized to the Ethiopian setting and pretested prior to actual data collection to ensure its clarity, validity, and reliability.

Qualitative data were collected using a semi-structured in-depth interview guide developed based on study objectives and relevant literature. The guide explored perceptions of the theory–practice gap, institutional factors, and instructor-related challenges affecting integration of theory and practice. Interviews were conducted face-to-face in private rooms within the institutions to ensure confidentiality and minimize interruptions. All interviews were conducted by the principal investigator, audio-recorded with consent, and supplemented with field notes.

### Timing between data collection phases

Quantitative data collection and preliminary analysis were completed first. Qualitative data collection was then conducted to further explore and explain key findings from the quantitative phase. This sequential timing facilitated the integration of results during interpretation to enhance our understanding of the theory–practice gap.

### Variables

The dependent variable was the theory–practice teaching gap. The independent variables included student-related factors (age, sex, clinical placement area, and motivation), instructor-related factors (communication between theoretical and clinical instructors, teaching experience, and continuity of teaching), and organizational factors (availability of learning resources, supervision, institutional support, and type of hospital), where these variables were selected based on evidence from previous studies.

### Data analysis

Quantitative data were entered into EpiData and analyzed using SPSS version 26 (IBM, Armonk, NY, USA). Descriptive statistics such as frequencies, percentages, means, and standard deviations were used. Binary logistic regression analysis was performed to identify factors associated with the theory–practice gap. Variables with *p*-values <0.05 were considered statistically significant.

The internal consistency of the questionnaire was assessed using Cronbach's alpha, which yielded a value of 0.83, indicating good reliability.

Qualitative data were analyzed using thematic analysis. Audio-recorded interviews were transcribed verbatim, followed by coding, categorization, and development of themes and subthemes.

### Trustworthiness and quality assurance

For the quantitative component, data quality was ensured through pretesting, training data collectors, and close supervision. Reliability was confirmed using Cronbach's alpha.

For the qualitative component, credibility was ensured through member checking and prolonged engagement. Dependability and confirmability were maintained through systematic documentation of the coding and analysis procedures.

### Data integration and triangulation

The quantitative and qualitative findings were integrated during the interpretation stage using triangulation. Quantitative data provided the magnitude of the theory–practice gap and associated factors, and the qualitative findings helped to explain and contextualize these results. Convergence and complementarity between data sets were assessed to strengthen validity and provide a comprehensive understanding of the results.

## Results

In total, 422 undergraduate nursing students participated in the study, where most were female (82.7%, n = 349) and nearly half were aged 23–27 years (49.3%, n = 208). Most participants were assigned to teaching hospitals (86.7%, n = 366), whereas 13.3% (n = 56) practiced in non-teaching hospitals. The majority of students were placed in maternity wards (39.3%, n = 166) (Table 1).

**Table 1:** Socio-demographic characteristics of study participants.

Variables	Frequency	%
**Age category**		
18–22 years	208	49.3
23–27 years	207	49
≥28 years	7	1.7
**Sex**		
Male	73	17.3
Female	349	82.7
**Types of hospital**		
Teaching hospital	366	86.7
Non-teaching hospital	56	13.3
**Types of wards**		
Surgical ward	20	4.7
Maternity ward	166	39.3
Medical ward	84	19.9
Emergency ward	84	19.9
Pediatric ward	68	16.1

**Figure 1:**
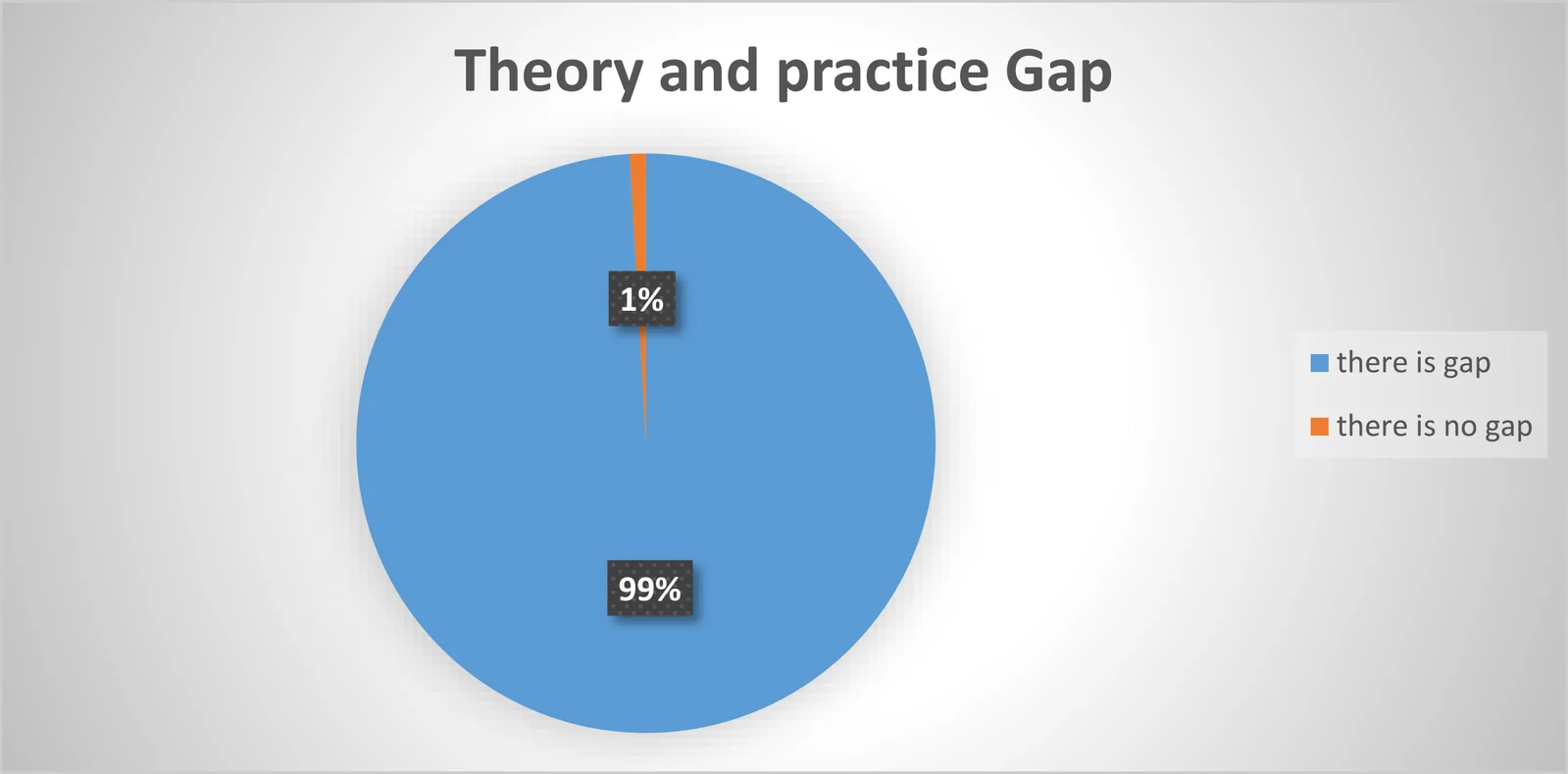
Showing level of theory and practice teaching gap of study participants at west and south west shoa higher teaching institution, 2024.

Almost all participants (99.1%, n = 418) reported the existence of a theory–practice gap. The overall mean score for the theory–practice gap was 3.6, and only 0.9% (n = 4) scored below the mean (Figure 1). In terms of institutional factors, the mean score for provision of theoretical teaching was 3.2, and 94.1% (n = 397) of participants scored above the mean. In addition, 41.2% (n = 174) agreed that their institution provided adequate learning resources, whereas 14.0% (n = 59) disagreed (Table 2). In terms of instructor-related factors, 27.5% (n = 117) reported inadequate communication between theoretical and clinical instructors, whereas 26.5% (n = 112) disagreed. Furthermore, 38.2% (n = 161) of clinical practice was not fully consistent with classroom learning (Table 3).

**Table 2:** Organizational factors contributing to the theory–practice gap in nursing education.

Question		SDA	DA	N	A	SA	Mean	Standard deviation
Does the college provide educational opportunities and sources of self-learning such as the internet or a library?	**Freq.**	24	59	83	174	82	3.54	1.22
	**%**	5.7	14	19.7	41.2	19.4		
Are the classroom and labs appropriate and equipped with audio and visual aids, and lab supplies?	**Freq.**	26	75	125	112	84	3.4	1.2
	**%**	6.2	17.8	29.6	26.5	19.9		
Is the college developing your learning skills such as communication and discussion?	**Freq.**	26	75	125	112	84	3.4	1.2
	**%**	6.2	17.8	29.6	26.5	19.9		
Is the college working with you to promote and develop your self-confidence?	**Freq.**	47	111	82	135	47	3.05	1.21
	**%**	11.1	26.3	19.4	32	11.1		
Is the college making you aware of the legal and ethical standards of the profession?	**Freq.**	76	72	82	142	50	3.04	1.31
	**%**	18	17.1	19.4	33.6	11.8		
Does the simulation lab help you to develop a deeper understanding of being a nurse?	**Freq.**	72	135	78	112	25	2.72	1.2
	**%**	17.1	32	18.5	26.5	5.9		
Do you have sufficient time in theory and practical lectures?	**Freq.**	23	132	82	126	59	3.2	1.2
	**%**	5.5	31.3	19.4	29.9	14		

SDA = strongly disagree; DA = disagree; N = neutral; A = agree; SA = strongly agree.

**Table 3:** Instructor-related factors contributing to the theory–practice gap in nursing education.

Question		SDA	DA	N	A	SA	Mean	Standard deviation
There is a lack of communication between theoretical teachers and clinical teachers	**Freq.**	80	112	83	116	31	2.8	1.24
	**%**	19	26.5	19.7	27.5	7.3		
Instructors do not consider differences between students	**Freq.**	43	106	70	122	81	3.2	1.3
	**%**	10.2	25.1	16.6	28.9	19.2		
When you practice in the ward, do you feel that we do not apply all that we learn in theoretical class?	**Freq.**	11	41	135	132	103	3.7	1.03
	**%**	2.6	9.7	32	31.3	24.4		
Does your clinical instructor have poor communication with you when you practice in hospital?	**Freq.**	2	60	101	156	103	3.7	1.005
	**%**	0.5	14.2	23.9	37	24.4		
Does the training in the practice class continue what you had already learned in the theoretical class?	**Freq.**	24	88	95	161	54	3.32	1.11
	**%**	5.7	20.9	22.5	38.2	12.8		

SDA = strongly disagree; DA = disagree; N = neutral; A = agree; SA = strongly agree.

According to multivariate logistic regression analysis, type of hospital was significantly associated with the theory–practice gap. Students assigned to non-teaching hospitals were 2.83 times more likely to experience a theory–practice gap compared with those in teaching hospitals (adjusted odds ratio = 2.829; 95% confidence interval: 1.428–5.602; *p* = 0.003). The qualitative findings identified two main themes: university/college focus on theoretical teaching and university/college focus on clinical teaching (Table 4). Participants described variability in teaching approaches, curriculum imbalance, limited institutional collaboration, inadequate clinical preparation, and challenges applying theoretical knowledge in clinical settings. They also reported limited educator involvement in clinical environments and gaps in coordination between academic and hospital settings (see Table 5).

**Table 4:** Themes and sub-themes related to theoretical teaching.

Major theme	Sub-themes	Analytic codes
University focus on theoretical teaching	Students' satisfaction with theoretical learning	Perceived satisfaction, indirect evaluation, lack of feedback, workload, motivation
	Instructors' teaching experience	Active learning methods, limited training, reliance on lecture
	Importance of courses	Core vs. supportive courses, curriculum balance
	Curriculum relevance and need for modification	Missing content, imbalance, ongoing revision
	Training and implementation of problem-based learning	Student-centered learning, institutional support, time limitations

**Table 5:** Themes and sub-themes related to clinical teaching.

Major theme	Sub-themes	Analytic codes
University/college focus on clinical or hospital teaching	First experience in clinical teaching	Initial fear, emotional adjustment, ethical challenge, transition to practice
	Institutional disconnection and resource constraints	University–hospital gap, limited resources, restricted educator role
	Lack of clinical preparedness and support	Inadequate preparation, reliance on prior experience, self-directed learning
	The theory–practice gap	
–	The theory–practice gap	Knowledge–practice mismatch, curriculum misalignment, poor integration
	Educator roles and professional identity	Underutilized expertise, weakened identity, need for collaboration

## Implications for curriculum development, faculty training, and clinical integration strategies to bridge the theory–practice gap

The findings obtained in this study emphasize the urgent need for curriculum reforms that explicitly integrate theoretical knowledge with clinical practice through competency-based learning, simulation-based training, case-based learning, and early structured clinical exposure. Faculty development programs should focus on strengthening both clinical competency and mentorship skills to enhance effective bedside teaching. Strong institutional partnerships between universities and hospitals are essential for improving supervision, coordination, and student learning experiences. Adequate resource allocation, continuous monitoring of clinical competence, and shared clinical learning platforms can significantly reduce the theory–practice gap and improve the quality of nursing education and patient care.

## Discussion

This study aimed to assess the theory–practice gap in undergraduate nursing education and identify the underlying factors in selected teaching institutions in the west and southwest Shewa Zones. The findings indicated a substantial gap between theoretical instruction and clinical practice, where nearly all participants reported inconsistencies between classroom learning and clinical practice learning, thereby highlighting a persistent challenge in applying theoretical knowledge to clinical competence.

These findings are consistent with those obtained in previous studies conducted in Lahore, Pakistan, indicating a clear gap between theoretical and practical components of nursing education, which was largely attributed to poor integration between classroom and clinical training, and low student self-confidence.^12^ Similar challenges have been reported in Namibia, where nursing students experienced difficulties applying theoretical knowledge in clinical settings.^3^ Evidence from Pakistan further indicates that a substantial proportion of nurses had poor knowledge regarding theory–practice integration, reflecting their limited awareness and application of theoretical concepts in practice. Overall, these findings suggest that the theory–practice gap may be more common in low- and middle-income countries, possibly due to systemic constraints, such as limited educational resources, inadequate clinical infrastructure, and insufficient support for practice-based learning.

## Institutional and instructor-related factors

Institutional and instructor-related factors were identified as significant contributors to the theory–practice gap. Limited availability of learning resources, restricted access to self-directed learning tools such as libraries and internet services, and insufficient opportunities for practical skill development appeared to hinder the effective integration of theory into practice. These findings are consistent with evidence from Iran highlighting the critical roles of supervision, evaluation, and structured educational processes in minimizing the theory–practice gap in nursing education.^11^ The observed similarities may reflect broader systemic challenges that are common in developing countries, where constraints on human resources and educational infrastructure limit the quality and effectiveness of clinical teaching.

Students generally perceived that universities provided some level of practice-based teaching, as reflected by the moderate mean score and mainly positive responses. This finding is comparable to a study conducted in the Gaza Strip, Palestine, which also reported moderate to high student satisfaction with clinical teaching. However, despite this apparent emphasis on practice, the findings obtained in the present study indicate that clinical training may lack sufficient depth and effective integration with theoretical instruction.^4^ This suggests that the issue is not due to the availability of practice opportunities, but instead to the quality, consistency, and alignment of clinical teaching with classroom learning.

## Qualitative insights: emotional and ethical challenges in clinical teaching

The qualitative findings highlighted the significant emotional and ethical challenges experienced by both students and instructors during clinical practice. Participants frequently reported feelings of fear, confusion, and lack of confidence when transitioning into clinical environments, particularly when managing critically ill patients. In addition, ethical dilemmas and difficulties performing advanced clinical procedures were commonly described, indicating gaps in technical competence but also in ethical preparedness.

These findings are consistent with a study conducted in Jordan, where clinical instructors were reported to have limited practical experience despite their strong theoretical knowledge, thereby constraining their ability to effectively bridge theory and practice. Consequently, clinical teaching often remained predominantly theory-oriented, even within practical settings.^1^ This suggests that insufficient clinical competency among instructors may contribute to ineffective skill transfer and limit the ability of students to apply theoretical knowledge in clinical settings.

Participants in the present study emphasized that although clinical practice provided valuable real-world exposure, it was often not standardized, leading to variability in student learning experiences. Poor communication between theoretical and clinical instructors further compounded this issue, resulting in inconsistent supervision and assessment across training sites. These challenges undermined the continuity and coherence of clinical education and limited the effective integration of theory into practice.

## Limitations of the study

This study did not involve observations of teaching theory and practice.

Researcher bias: data collection and interpretation relied heavily on the researcher's perspectives, which may have influenced how responses were probed, coded, and interpreted.

Subjectivity of data: the responses of participants were shaped by their personal feelings, experiences, and context, which may have made the findings less “objective.”

Bias due to exclusion of certain groups: instructors who did not deliver major courses may have been intentionally excluded.

## Conclusion and recommendation

This study identified a substantial theory–practice gap in nursing education. The quantitative findings showed that most students reported the existence of a gap and identified institutional factors such as type of hospital and limited learning resources as significant contributors. The qualitative findings indicated institutional disconnection between universities and hospitals, inadequate clinical preparation, and weak communication between theoretical and clinical instructors. Triangulating these findings showed that both structural and educational factors contributed to the persistence of the theory–practice gap. Addressing these issues requires stronger collaboration between academic institutions and clinical facilities, improved clinical supervision, and better integration of theoretical teaching with practical training.

Therefore, higher education institutions in the west and southwest Shewa Zones should strengthen both theoretical and clinical teaching by increasing the number of qualified clinical instructors, as well as improving communication between theoretical and clinical educators. Universities should organize regular training workshops to enhance clinical teaching skills and bridge the theory–practice gap. In addition, stronger collaboration between universities and healthcare institutions should be established in line with Ministry of Health and Ministry of Education standards to improve clinical learning environments and ensure the effective integration of theory and practice.

## Ethical approval

Ethical approval was obtained from Ambo University Institutional Review Board. Informed consent was obtained from all participants prior to data collection. Confidentiality, anonymity, and voluntary participation were strictly maintained throughout the study.

## Authors contributions

SAW: Project administration, Funding acquisition, Writing - original draft; Writing - review & editing. AD: Conceptualization; Data curation. MRT: Formal analysis; Investigation. KD: Methodology; Software; Supervision; Validation; Visualization. All authors have critically reviewed and approved the final draft and are responsible for the content and similarity index of the manuscript.

## Declaration of generative AI and AI-assisted technologies in the manuscript preparation process

During the preparation of this work, the authors used ChatGPT in order to correct some spelling and grammar issues, especially in the Results section. After using this tool, the authors reviewed and edited the content as needed, and take full responsibility for the content of the published article.

## Source of funding

This work was supported by the Ambo University College of Health Sciences and Referral Hospital.

## Conflict of interest

The authors have no conflict of interest to declare.
